# Haplotypes of the porcine peroxisome proliferator-activated receptor delta gene are associated with backfat thickness

**DOI:** 10.1186/1471-2156-10-76

**Published:** 2009-11-30

**Authors:** Karina Meidtner, Hermann Schwarzenbacher, Maren Scharfe, Simone Severitt, Helmut Blöcker, Ruedi Fries

**Affiliations:** 1 Chair of Animal Breeding, Technical University of Munich, Hochfeldweg 1, 85354 Freising - Weihenstephan, Germany; 2 Department of Genome Analysis, Helmholtz Centre for Infection Research, Inhoffenstraße 7, 38124 Braunschweig, Germany

## Abstract

**Background:**

Peroxisome proliferator-activated receptor delta belongs to the nuclear receptor superfamily of ligand-inducible transcription factors. It is a key regulator of lipid metabolism. The peroxisome proliferator-activated receptor delta gene (*PPARD*) has been assigned to a region on porcine chromosome 7, which harbours a quantitative trait locus for backfat. Thus, *PPARD *is considered a functional and positional candidate gene for backfat thickness. The purpose of this study was to test this candidate gene hypothesis in a cross of breeds that were highly divergent in lipid deposition characteristics.

**Results:**

Screening for genetic variation in porcine *PPARD *revealed only silent mutations. Nevertheless, significant associations between *PPARD *haplotypes and backfat thickness were observed in the F2 generation of the Mangalitsa × Piétrain cross as well as a commercial German Landrace population. Haplotype 5 is associated with increased backfat in F2 Mangalitsa × Piétrain pigs, whereas haplotype 4 is associated with lower backfat thickness in the German Landrace population. Haplotype 4 and 5 carry the same alleles at all but one SNP. Interestingly, the opposite effects of *PPARD *haplotypes 4 and 5 on backfat thickness are reflected by opposite effects of these two haplotypes on PPAR-δ mRNA levels. Haplotype 4 significantly increases PPAR-δ mRNA levels, whereas haplotype 5 decreases mRNA levels of PPAR-δ.

**Conclusion:**

This study provides evidence for an association between *PPARD *and backfat thickness. The association is substantiated by mRNA quantification. Further studies are required to clarify, whether the observed associations are caused by *PPARD *or are the result of linkage disequilibrium with a causal variant in a neighbouring gene.

## Background

In various pig populations genome wide studies have been carried out to map quantitative trait loci (QTL) and to develop genetic markers for breeding. This resulted in more than 400 QTL for fatness [1]. The numerous QTL studies revealed chromosomal regions repeatedly linked to fatness traits. Some of the most significant QTL for backfat were identified on porcine chromosome 7 [2-5]. Most studies report a paradox of lower backfat to be caused by the allele originating from the breed, usually Meishan, with the higher backfat mean [2,5-7]. In this QTL region the peroxisome proliferative activated receptor delta (PPAR-δ) gene was mapped [8,9]. PPAR-δ is involved in the regulation of lipid metabolism, energy balance and insulin sensitivity [10]. Therefore, *PPARD *is considered to be a functional as well as positional candidate gene for backfat thickness. A study of 74 porcine SNPs across 5 chromosomes, with the majority located in proximity to backfat QTL, revealed an association of two markers in *PPARD *with backfat thickness [11].

The metabolic and histochemical characterisation of fat and muscle tissue from pigs with the chromosome 7 QTL alleles from Meishan and Large White showed differences in adipocyte size and number in backfat as well as differences in the basal rate of glucose incorporation into lipids and activity of lipogenic enzymes [12]. Therefore, *PPARD *is considered to be a promising candidate gene for the observed QTL effect [12]. A microarray-based experiment aiming at the identification of differentially expressed genes between lean Piétrain and 'obese' German Landrace pigs revealed an up-regulation of *PPARD *in Piétrain [13]. This suggests a possible effect on lipid deposition and strengthens the hypothesis of *PPARD *being a candidate gene for fatness.

The objective of this study was to systematically screen the PPAR-δ gene for polymorphisms and carry out association studies with identified variants. Furthermore, mRNA expression of *PPARD *variants was analysed in liver.

## Results

### Gene structure, splice variants and their expression

**Figure 1:**
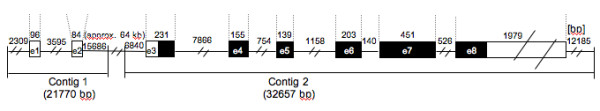
**Exon-Intron structure of *PPARD***.

The genomic structure of the porcine *PPARD *was previously unknown. Therefore, it had to be determined from the porcine RefSeq mRNA sequence that consists of eight exons [GenBank: NM_214152]. Genomic sequence data was obtained by sequencing a porcine BAC (PigE-255B24). The assembly of the BAC shot gun sequences resulted in two genomic contigs containing *PPARD*. Sequence data was submitted to gene bank [GenBank: EU169095]. The first contig comprises the putative promoter, exon 1, the complete intron 1, exon 2 and approximately 15.7 kb of intron 2 (Figure 1). The second contig covers the region from exon 3 to exon 8 and 12.2 kb of the region 3' of the last exon (Figure 1). The resulting *PPARD *mRNA sequence is complete. All introns follow the GT-AG rule. The protein-coding sequence starts in exon 3 and ends in exon 8. The derived amino acid sequence is 94.6% identical to the sequence of human PPAR-δ. The common PPAR-δ transcript includes all eight exons. In addition, a splice variant without exon 2 was detected. Both PPAR-δ splice variants seem to be ubiquitously expressed since they were detected in liver, lung, backfat, muscle, kidney, brain, spleen and heart tissue.

### Polymorphisms and haplotypes

All eight exons of porcine *PPARD*, exon flanking intronic regions and 2000 bp of the putative promoter region were screened for genetic variation by resequencing the parental generation of a Mangalitsa × Piétrain intercross and three unrelated animals of each of the German Landrace, German Large White and Duroc breeds. A total of 25 variants were identified, comprising 22 SNPs, two insertion/deletion polymorphisms and one stretch of a variable number of cytosins (Table 1). Two out of 22 SNPs are located in the protein-coding region, but they do not change the amino acid sequence. The number of cytosins in the polyC stretch varied between 11 and 14 Cs in the analysed animals. However, it was impossible to determine the exact number of Cs of the polyC stretch in some heterozygous animals. For that reason, only 24 polymorphisms were used to infer haplotypes.

**Table 1:** Polymorphisms in *PPARD*

dbSNP number	Variant	Localisation	Predicted function
ss161109991	SNP	Promoter	unknown
ss161109992	SNP	Promoter	unknown
ss161109993	SNP	Promoter	unknown
ss161109994	SNP	Promoter	unknown
ss161109995	SNP	Promoter	affects putative ETS-domain TFBS
ss161109996	SNP	Intron 1	unknown
ss161109997	SNP	Exon 2 (5' UTR)	Minor effect on mRNA structure
ss161109998	SNP	Exon 2 (5' UTR)	Minor effect on mRNA structure
ss161109999	SNP	Intron 3	unknown
ss161110000	SNP	Intron 4	unknown
ss161110001	SNP	Intron 4	unknown
ss161110002	SNP	Intron 5	unknown
ss161110003	InDel	Intron 5	unknown
ss161110004	SNP	Intron 5	unknown
ss161110005	SNP	Intron 5	unknown
ss161110006	SNP	Intron 5	unknown
ss161110007	SNP	Intron 6	unknown
ss161110008	InDel	Intron 6	unknown
ss161110009	SNP	Exon 7 (coding)	synonymous, minor effect on mRNA structure
ss161110010	SNP	Exon 8 (coding)	synonymous, minor effect on mRNA structure
ss161151656	SNP	Exon 8 (3' UTR)	minor effect on mRNA structure
ss161151657	SNP	Exon 8 (3' UTR)	no effect on mRNA structure
ss161151658	SNP	Exon 8 (3' UTR)	no effect on mRNA structure
ss161151659	SNP	Exon 8 (3' UTR)	minor effect on mRNA structure
ss161151660	polyC	Exon 8 (3' UTR)	minor effect on mRNA structure

A total of five haplotypes were detected with haplotypes 4 and 5 being identical at 23 out of 24 polymorphisms (Table 2). Both founding Mangalitsa boars of the resource population were homozygous for haplotype 1. The haplotype frequencies were 17%, 46%, 17%, 8% and 12% for haplotype 1, 2, 3, 4 and 5, respectively, in the twelve Piétrain parental animals. Four SNPs (polymorphisms ss161109995, ss161109998, ss161110009 and ss161110010) are sufficient to tag the five haplotypes.

**Table 2:** Haplotypes of *PPARD*

SNP	*PPARD *Haplotype				
	1	2	3	4	5
**ss161109991**	A	A	A	G	G
**ss161109992**	G	G	A	G	G
**ss161109993**	A	A	G	G	G
**ss161109994**	C	C	C	T	T
**ss161109995**	A	A	A	C	C
**ss161109996**	A	A	G	G	G
**ss161109997**	C	C	C	T	T
**ss161109998**	G	G	A	A	A
**ss161109999**	G	G	A	G	G
**ss161110000**	T	T	T	C	C
**ss161110001**	G	G	G	A	A
**ss161110002**	C	C	T	C	C
**ss161110003**	I	I	I	D	D
**ss161110004**	T	C	C	C	C
**ss161110005**	A	G	G	G	G
**ss161110006**	A	A	A	G	G
**ss161110007**	C	C	C	T	T
**ss161110008**	D	D	D	I	I
**ss161110009**	G	G	G	G	A
**ss161110010**	G	T	T	T	T
**ss161151656**	G	G	T	G	G
**ss161151657**	C	T	C	C	C
**ss161151658**	T	C	T	T	T
**ss161151659**	T	C	C	C	C

D - deletion, I - insertion

### Association studies

Association between *PPARD *haplotypes and backfat thickness was initially studied in the F2 generation of a Mangalitsa × Piétrain cross. Analysis of haplotype frequencies in the F1 generation revealed that haplotype 4 was not passed on to the F1 generation and was consequently assumed to be absent in the F2 generation. Therefore, SNPs ss161109995, ss161109998 and ss161110010 were sufficient to distinguish the remaining four haplotypes and were used to genotype 599 F2 animals by diagnostic restriction enzyme assays. Association analyses of *PPARD *variants and backfat were carried out and revealed a significant association between *PPARD *haplotype 5 and backfat thickness (p = 0.022, Table 3) in the Mangalitsa × Piétrain cross when contrasted against all other haplotypes. However, the p-value is above the 5% bonferoni corrected significance threshold of 0.0125 that corrects for testing of four different haplotypes. Heterozygous animals carrying one haplotype 5 allele showed an increase in backfat thickness of 2.43 mm (Table 3).

**Table 3:** Association analysis of *PPARD *haplotypes and backfat thickness in the middle of the back

Breed	Haplotype	Number of animals			Haplotype LS mean [mm] (SE)			P-value
		-/-	+/-	+/+	-/-	+/-	+/+	
F2 Mangalitsa × Piétrain	1	97	280	222	29.50 (0.56)	29.29 (0.32)	29.16 (0.37)	0.902
	2	300	247	52	29.30 (0.33)	29.14 (0.35)	29.80 (0.81)	0.744
	3	522	77	-	29.38 (0.24)	28.57 (0.72)	-	0.311
	5	553	46	-	29.09 (0.23)	31.52 (1.00)	-	0.022

German Landrace	1	544	124	7	20.56 (0.16)	20.96 (0.33)	18.76 (1.39)	0.220
	2	193	366	116	20.37 (0.27)	20.63 (0.19)	20.96 (0.34)	0.388
	3	579	95	1	20.57 (0.15)	20.92 (0.38)	16.01 (3.67)	0.320
	4	271	314	90	20.86 (0.22)	20.64 (0.21)	19.78 (0.38)	0.034
	5	654	19	2	20.58 (0.14)	21.92 (0.85)	19.00 (2.60)	0.242

**Figure 2:**
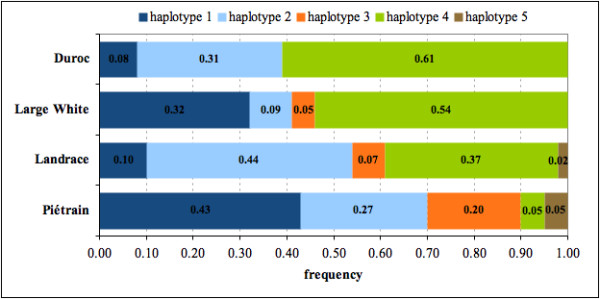
**Frequency of *PPARD *haplotypes indifferent pig breeds**.

This result was followed up in unrelated animals of a commercial pig breed. The selection of a suitable study population was problematical because haplotype 5 is infrequent in all analysed pig populations (Figure 2). The highest frequency was observed in Piétrain and is estimated at 5% (Figure 2). Nevertheless, haplotype 4 is identical to haplotype 5 at all but one SNP (ss161110009, Table 2) and haplotype 4 is frequent in German Landrace, German Large White and Duroc (Figure 2). German Landrace was chosen because it was expected to exhibit both a relatively high frequency of haplotype 4 and possibly a few animals carrying haplotype 5. A total of 681 animals were successfully genotyped. The frequencies were 10%, 44%, 7%, 37% and 2% for haplotypes 1, 2, 3, 4 and 5, respectively. Genotype frequency of all tag SNPs did not deviate from Hardy-Weinberg equilibrium. Backfat thickness was significantly decreased by *PPARD *haplotype 4 (p = 0.034) when tested against all other haplotypes. In contrast, haplotype 5 that differs only in one SNP from haplotype 4 increased backfat thickness in the Mangalitsa × Piétrain population (Table 3). Haplotype 5 has no significant effect on backfat thickness (p = 0.242) in the German Landrace population. The power to detect a significant association (p < 0.05) of the rare haplotype 5 (MAF = 2%) within the analysed German Landrace population of 700 individuals is only 0.05. Therefore, we cannot exclude an effect of haplotype 5 on backfat thickness in this population. The least square means of backfat thickness within the group of pigs carrying one haplotype 5 allele is higher than in pigs carrying no haplotype 5 (Table 3), which is the same trend as seen in the Mangalitsa × Piétrain population. Least square means of pigs homozygous for haplotype 5 cannot be reliably estimated since there are only two animals in that group.

### Expression of *PPARD *variants

Our association studies suggest evidence for association between *PPARD *variants and backfat thickness. However, since two different haplotypes were associated with an opposing effect on backfat thickness in two different pig populations, it is unclear if the observed associations are caused by a variation in *PPARD *or a causal mutation in linkage disequilibrium to *PPARD*. None of the SNPs located in the coding region cause an amino acid exchange. For this reason, there is no obvious functional candidate for the observed association. However, numerous studies have identified cis-regulatory and synonymous mutations with functionally significant consequences for morphology, physiology and behaviour [14-16]. To estimate whether the detected polymorphisms in *PPARD *could be functional, gene expression of the *PPARD *haplotypes 4 and 5 was studied. Pigs containing the desired *PPARD *variants were bred by artificial insemination of F3 sows with Piétrain boars known to possess haplotype 4 and haplotype 5, respectively. This was necessary since none of the F3 animals possessed haplotypes 4 and 5. Consequently, offspring carrying no or one haplotype 4 or 5 allele was obtained. Tissue samples from liver were collected within 30 min after exsanguation and stored in RNAlater™ (Qiagen, Hilden, Germany). RNA was isolated, reverse transcribed and PPAR-δ expression was analysed in a relative quantification approach with Tata-Box binding protein (TBP) and Topoisomerase 2 beta (TOP2B) as reference genes. PPAR-δ expression is significantly reduced by haplotype 5 and increased by haplotype 4 (Table 4). Interestingly, haplotypes 4 and 5 are the two haplotypes associated with backfat thickness. The presence of haplotype 5 increased backfat thickness in a Mangalitsa × Piétrain cross and the presence of haplotype 4 decreased backfat thickness in a German Landrace population. In accordance with these findings, PPAR-δ expression is altered in opposite directions by these two haplotypes.

**Table 4:** mRNA expression of PPAR-δ porcine liver

Haplotype	number of animals		PPAR-δ expression	
	-/-	+/-	R	p-value
1	11	42	0.47	0.044
2	43	10	1.69	0.182
3	35	18	1.14	0.704
4	32	21	2.13	0.014
5	41	12	0.35	0.004

Ratios indicate the influences of a *PPARD *haplotype on mRNA levels. Ratio R = 1 indicates no influence, R > 1 indicates mRNA levels are increase by the haplotype and R < 1 indicates a decrease. P-values were estimated by a permuation method.

Statistical significant reduced expression was reached by haplotype 1 as well. However, in the analysis absence of haplotype 1 was compared with mostly heterozygous presence of haplotype 1. Further analysis of PPAR-δ expression in animals with diplotypes containing haplotype 1 showed a reduced expression only in animals carrying diplotype 1/5, which is most likely caused by haplotype 5.

## Discussion

*PPARD *was chosen as a candidate for backfat thickness because of the central role of PPAR-δ in the regulation of lipid metabolism and because of its localisation in a major QTL region for backfat thickness on chromosome 7. The candidate gene analysis of porcine *PPARD *presented here identified only silent mutations. Nonetheless, it reveals an association of *PPARD *haplotypes 4 and 5 with backfat thickness. Haplotype 5 is associated with increased backfat thickness in F2 Mangalitsa × Piétrain pigs, and haplotype 4 with decreased backfat thickness in the German Landrace population. It was not possible to carry out an association study with haplotypes 4 and 5 in the same pig population, as haplotype 4 is absent in the F2 Mangalitsa × Piétrain generation and haplotype 5 is extremely rare in German Landrace. Analogous to numerous QTL studies [2,5-7], the same paradox of lower backfat caused by the allele originating from the breed with more backfat was revealed. In the Mangalitsa × Piétrain cross, haplotype 5 originates from the lean Piétrain breed and causes higher backfat.

Interestingly, the opposite effect of *PPARD *haplotypes 4 and 5 on backfat thickness is reflected by an opposite effect of these two haplotypes on PPAR-δ mRNA levels. Haplotype 4 is associated with reduced backfat thickness, and it significantly increases mRNA expression of PPAR-δ in liver. Haplotype 5 is associated with higher backfat thickness, and it significantly decreases PPAR-δ expression in liver. These findings are in line with studies demonstrating a decrease of body fat in mice caused by PPAR-δ overexpression [17]. In conclusion, findings from the association study, when considered together with results from the PPAR-δ expression study may suggest an effect of *PPARD *variants on backfat thickness. However, this study was not able to verify that, because no obvious functional candidate was identified. None of the SNPs located in the coding sequence result in an amino acid exchange. None of the SNPs in the analysed 2000 bp region of the *PPARD *promoter are located in a conserved region or a region that is known to harbour a transcription factor binding site [18]. Differences in mRNA levels between *PPARD *haplotypes suggest that functionality is caused either by influences on mRNA stability or by differences in PPAR-δ mRNA de-novo synthesis. None of the SNPs located in mRNA exhibit a large influence on the mRNA secondary structure (data not shown). However, control elements located in introns or far away from the gene can enhance or inhibit mRNA expression. This makes it difficult to identify the functional variant, especially as the observed effect might be due to not only one, but several genetic variants interacting with each other. In conclusion, this study was not able to detect a genetic variant in *PPARD *that is likely to cause the observed association. Hence, the effect on backfat thickness can originate from another variant in linkage disequilibrium to the analysed haplotypes located either in *PPARD *or a neighbouring gene.

## Conclusion

The candidate gene study involving *PPARD *revealed association between *PPARD *variants and backfat thickness. However, it is not clear whether or not the association is caused by *PPARD *or a neighbouring gene in linkage disequilibrium, especially since no obvious functional variant was identified. Further studies are required to determine whether the observed associations are present in other pig populations. Additionally, other candidate genes at the location of the QTL on porcine chromosome 7 should be considered.

## Methods

### Animals

#### Mangalitsa × Piétrain intercross

Initially, two Swallow Bellied Mangalitsa boars were crossed to 13 Piétrain sows to produce an F1 generation. All Piétrain sows were homozygous for the mutant Cys614 allele at the *RYR1 *locus. Selected F1 sows (n = 18) and boars (n = 5) were mated and the resulting F2 generation was used for the analysis presented here. Animals were fed ad libitum. Male pigs were castrated. Pigs were slaughtered when they reached a weight of about 95 kg. Backfat thickness at the middle of the back was measured after slaughtering according to German Pig Breeders Standards [19]. A mean backfat thickness of 29.27 mm with a standard deviation of 5.57 mm was observed.

Association analyses were carried out in the F2 generation. F3 sows were backcrossed with Piétrain boars to introduce a desired *PPARD *gene variant (haplotypes 4 and 5) that was lost in F3. The genotypes of Piétrain boars were determined in order to assure the presence of the *PPARD *haplotypes 4 and 5 in the employed Piétrain boars. Seven F4 litters were used for gene expression studies. These seven litters correspond to a total number of 56 animals, 31 of them male and 25 female. They were slaughtered at 80 ± 2 days of age with an average weight of 25.6 kg. Tissue samples (approximately 0.5 g) for RNA isolation were collected from liver, longissimus muscle, backfat, heart, spleen, brain, kidney, ham and lung within 30 min after exsanguination and stored in 5 ml of RNAlater™ (Qiagen, Hilden, Germany).

#### Commercial Pig Breeds

A total of 722 German Landrace pigs raised under standardized condition for performance testing between 2002 and 2005 in Bavaria were used for association analysis. Backfat measurement was carried out after slaughtering according to German Pig Breeders Standards [19]. A mean backfat thickness of 20.64 mm with a standard deviation of 3.78 mm was observed.

The estimation of allele frequencies in different breeds was performed in 213 Piétrain, 40 German Landrace, 13 Large White, 24 Meishan and 45 Duroc animals.

### BAC clone

A bacterial artificial chromosome (BAC) clone containing *PPARD *was identified *in-silico *with the help of the genomic location of the human gene from the Porcine BAC End Sequencing Project [20]. Colony PCRs with primers located in the putative promoter region and in exon 8 (primer pair 2 and 15) were carried out to ensure that the clone contains *PPARD*. Sequencing of BAC PigE-255B24 was performed by a shotgun approach as follows: Sheared fragments of 3 kb in length (GeneMachines) were subcloned separately into pUC19 vector. 4 × 384 clones were selected from the clone library. Plasmid DNA was prepared following a protocol supplied by Millipore (Schwalbach, Germany). Cycle sequencing was routinely performed using ABI PRISM BigDye Terminator v 3.1. Ready Reaction Cycle Sequencing Kit (Applied Biosystems, Foster City, CA, USA) and M13f/M13r primer. All separations were run on ABI 3730XL capillary sequencers. Data were assembled and edited using the GAP4 program [21].

### DNA-Isolation, primer design, PCR, Re-sequencing

DNA was isolated from blood, semen and ear tissue by standard methods. Primers were designed with Primer 3 Software [22] based on porcine GSS available through the NCBI homepage [23] and on derived BAC sequences. The primers used for Polymerase Chain Reaction (PCR) are summarized in Table 5. A standard PCR reaction contained 0.5 μM of each Primer, 200 μM of each dNTP (Fermentas, St. Leon-Rot, Germany), 0.5 U Taq-Polymerase (Qiagen), 50 ng genomic DNA and the diluted 10-fold PCR buffer supplied by Qiagen (Tris/HCl buffer (pH = 8.7) containing 15 mM MgCl_2_, KCl, (NH_4_)_2_SO_4_) in a total volume of 20 μL. After preincubation at 94°C for 3 min, the PCR mixture underwent 30 cycles of denaturation at 94°C for 30 s, annealing for 60 s and extension at 72°C for 60 s. A final elongation step at 72°C for 3 min followed. The annealing temperature was adjusted to the requirements of the primers (Table 5). Cleaning of PCR products was undertaken in a MultiScreen^® ^PCRμ96 Plate (Millipore). The amount of cleaned PCR product used for sequencing reaction varied from 2 to 5.5 μL depending on fragment size and PCR efficiency. In addition to the PCR products, the sequencing reaction consisted of 2 μL reaction mix of the BigDye^® ^Terminator v1.1 Cycle Sequencing Kit (Applied Biosystems) and 0.5 μM primer. The volume of the sequencing reaction mix was adjusted to 10 μL. Thermal cycling for each primer was at 96°C for 10 s, 51°C for 5 s and 60°C for 4 min, for a total of 35 cycles. The sequencing reaction was cleaned via gel filtration with Sephadex G-50 (Sigma-Aldrich, Steinheim, Germany) in a MultiScreen^® ^96 well filtration plate (Millipore). DNA sequencing was performed on an ABI 377 automated sequencer (Applied Biosystems) according to manufactures instructions. Obtained sequences were analyzed using Phred/Phrap/Polyphred/Consed software suite [24-27].

**Table 5:** Primer sequences and PCR conditions

	localization	primer	annealing Tm [°C]
1	promoter	GAATGCCTCTTCCTGAATGG CCTCCTTGCCTTTGATATTGA	60

2	promoter	GGCAAGGAGGTTAACATCTGA GAGACTCCCCTGAATCACCA	60

3	promoter	GCAGCACAGTTTCCTCCAG GCTGCTTGCCTATCCACTTC	60

4	promoter, exon 1, intron 1	GGATTAATGGGAAAAGTTTTGG AGCAACTAACGACCGTGGAC	59

5	intron 1, exon 2	TCCAGGATTGAGAAAAATCTGC CAAGAATCCTAAACCTGGGATG	60

6	exon 3, intron 3	TCACCCTCTCATCCTCTACACC GCTGATTAGCGATAGAGTGACC	60

7	exon 4, intron4	CTGCCCCTGCTGTGTCTG AGGAAGAACCTACAAGCACCAC	65

8	intron 4	GCTTCCACTACGGAGTCCAC GATGAGGGAGGGTGAGAAAAG	59

9	exon 5	AACCATCTTTCTCCCTTCTTCG GCACTCCCTTCTGTCTCTGG	60

10	intron 5	GCTGGGCATGTCTCACAAC CAAAGCGAATGGCTGCATAG	59

11	exon 6, intron 6, exon 7	CTACAGCGCCTACCTGAAAAAC GAGAGCCAGGTCACTATCATCG	60

12	intron 5, exon 6, intron 6, exon 7	GCATCTCTGGGGAGTTCCTA TCGTTGAGGAAGAGGTGGTC	60

13	exon7, intron 7	TCTCTGTCTTTGCTCGTGTACC CCAGGAGGGCTGAGTGTG	67

14	intron 7	TAGTGACCTGGCTCTCTTCATC ATGGCCTCCACCTGTGAC	59

15	exon 8	CCAAGGTCCCCTGTCCTC GAGAGGAGGCAGGGCTATAAG	60

### Genotyping

Genotyping of tag SNPs was performed by PCR followed by restriction enzyme assays. For RFLP analysis, 3 - 7 μl of the appropriate PCR product were mixed with the enzyme and the supplied buffer. The volume was adjusted to 10 μl using water. An overview of enzymes used and reaction conditions employed is given in Table 6. After incubation at 37°C the resulting fragments were separated on a 2% agarose gel.

**Table 6:** Restriction enzymes

SNP	Enzyme	Specificity* 5' → 3'	Primer pair	Units per reaction	Incubation time
ss161109995	*Bsp143*II	PuG**C**GCPy	3	0.5	14 h
ss161109997	*Bsh1236*I	CG**C**G	5	0.5	14 h
ss161109998	*Eco72*I	CAC**G**TG	5	5	3 h
ss161110009	*Esp3*I	**C**GTCTC	12	0.5	14 h
ss161110010	*EcoO109*I	PuG**G**NCCPy	15	0.5	14 h

*polymorphic nucleotide marked in bold

### Bioinformatics

Transcription factor binding sites were predicted by Cister [28], P-Match [29] and MatInspector [30]. Prediction of mRNA secondary structure was carried out using the Mfold web server [31].

### Statistical analyses

Haplotypes were inferred using PHASE software version 2.1.1 [32,33] with default parameters (number of iterations = 100, thinning interval = 1, burn-in = 100).

Statistical analyses were carried out using the R environment for statistical computing version 2.4.1 [34]. Association between haplotypes and backfat thickness was estimated within 599 F2 Mangalitsa × Piétrain animals by a linear model with fixed effects of haplotype, dam and gender as well as covariate of living weight. For normalisation, backfat values were transformed to the power of 0.75. The model for estimating the effect of *PPARD *variants in the German Landrace population contained the performance testing station and weight as the only covariates because unrelated castrated animals were chosen. P-values were corrected for multiple testing of four haplotypes by Bonferroni correction. Least square (LS) means and their standard errors were calculated with untransformed backfat data based on the model described above using the effects package for R [35].

### Gene expression studies

Total RNA from 20 mg of RNAlater™ (Qiagen) stabilised liver tissue was isolated using the RNeasy^® ^Plus Mini Kit (Qiagen). Homogenisation of the tissue was achieved by processing the sample in the FastPrep^® ^Instrument (Qbiogene, Inc, CA, USA) for 40 seconds at a speed setting of 6.5 m/s using Lysing Matrix D (Qbiogene). Synthesis of cDNA was carried out for all samples at the same time with 1 μg RNA and 500 ng random pentadecamer primers using the First Strand cDNA Synthesis Kit (Fermentas). Quantitative Real-Time PCR was carried out on an ABI PRISM^® ^7000 Sequence Detection System (Applied Biosystems). Real-Time PCR reaction consisted of Power SYBR^® ^Green PCR Master Mix (Applied Biosystems), primers in an optimised concentration (*PPARD*: 100 nM 5'-CATGTCTCACAACGCCATTCG-3'/300 nM 5'-ATGTCGTGGATCACAAAGGGC-3'; TBP: 200 nM 5'-GATGGACGTTCGGTTTAGG-3'/300 nM 5'-AGCAGCACAGTACGAGCAA-3'; TOP2B: 200 nM 5'-GCTGGTGGCAAACACTCACTGG-3'/500 nM 5'-TGGAAAAACTCCGTATCTGTCTC-3') and diluted cDNA in a reaction volume of 20 μl.

After activation of Hot Start Polymerase by 10 min incubation at 95°C a 2-step PCR program was used consisting of 45 cycles of 15 s at 95°C and 1 min at 60°C. In case of *PPARD *annealing temperature had to be increased to 66°C to avoid primer dimers.

Crossing point (CP) and efficiency were calculated for each individual PCR reaction using ABI PRISM^® ^7000 SDS Software (Applied Biosystems) and the MoBPA package in R [36], respectively. For statistical analysis a modified version of the REST^© ^(Relative Expression Software Tool) method was applied [37]. The algorithm of REST^© ^allows group-wise comparison of relative expression data in Real-Time PCR. However, this method assumes equal amplification efficiencies in all samples. The method used here was adapted to account for differences in PCR efficiency.

Mean expression differences between different *PPARD *genotypes for all genes of interest were calculated as follows. In a first step, the expression E for each animal and each gene was determined (Equation 1).(1)

The geometric mean  of these expression values was calculated within a group of *N*_*gt *_animals with the same genotype *gt *(Equation 2).(2)

Finally, the mean expression of one genotype (*gt2*) was divided by the mean expression of the other genotype (*gt1*). For SNPs where 3 genotypes were present in the analysed animals the expression of one genotype (*gt1*) was compared to a pool of animals with the two other genotypes (*gt2*). The mean expression difference *R *was calculated by dividing the ratio for the gene of interest by the geometric mean of the ratios for *M *reference genes (Equation 3).(3)

A mean expression difference *R *= 1 characterises no effect of the genotype on expression of the analysed gene. The significance of a derivation from *R *= 1 was estimated by a permutation technique (number of permutations = 5000). The natural logarithm of *R *was used to obtain valid p-values for a two-sided significance test, because the untransformed values of *R *are left-skewed.

## List of abbreviations

BAC: Bacterial artificial chromosome; GSS: Genome survey sequence; LS: Least square; MAF: Minor allele frequency; PCR: Polymerase chain reaction; RFLP: Restriction Fragment Length Polymorphism; SNP: Single nucleotide polymorphism; QTL: Quantitative trait locus.

## Authors' contributions

KM carried out genetic studies, participated in statistical analysis and drafted the manuscript. HS participated in statistical analysis and edited the manuscript. RF designed the study and edited the manuscript. HB coordinated the BAC sequencing carried out by SS and MS. All authors read and approved the final manuscript.
